# The Effect of Active versus Passive Recovery Periods during High Intensity Intermittent Exercise on Local Tissue Oxygenation in 18 – 30 Year Old Sedentary Men

**DOI:** 10.1371/journal.pone.0163733

**Published:** 2016-09-27

**Authors:** Yuri Kriel, Hugo A. Kerhervé, Christopher D. Askew, Colin Solomon

**Affiliations:** 1 School of Health and Sports Sciences, University of the Sunshine Coast, Sippy Downs, QLD, Australia; 2 Laboratoire Interuniversitaire de Biologie de la Motricité, Université Savoie Mont Blanc, Le Bourget du Lac, France; Universita degli Studi di Roma 'Foro Italico', ITALY

## Abstract

**Purpose:**

High intensity interval training (HIIT) has been proposed as a time-efficient format of exercise to reduce the chronic disease burden associated with sedentary behaviour. Changes in oxygen utilisation at the local tissue level during an acute session of HIIT could be the primary stimulus for the health benefits associated with this format of exercise. The recovery periods of HIIT effect the physiological responses that occur during the session. It was hypothesised that in sedentary individuals, local and systemic oxygen utilisation would be higher during HIIT interspersed with active recovery periods, when compared to passive recovery periods.

**Methods:**

Twelve sedentary males (mean ± SD; age 23 ± 3 yr) completed three conditions on a cycle ergometer: 1) HIIT with passive recovery periods between four bouts (HIITPASS) 2) HIIT with active recovery periods between four bouts (HIITACT) 3) HIITACT with four HIIT bouts replaced with passive periods (REC). Deoxygenated haemoglobin (HHb) in the vastus lateralis (VL) and gastrocnemius (GN) muscles and the pre-frontal cortex (FH), oxygen consumption (VO_2_), power output and heart rate (HR) were measured continuously during the three conditions.

**Results:**

There was a significant increase in HHb at VL during bouts 2 (p = 0.017), 3 (p = 0.035) and 4 (p = 0.035) in HIITACT, compared to HIITPASS. Mean power output was significantly lower in HIITACT, compared to HIITPASS (p < 0.001). There was a significant main effect for site in both HIITPASS (p = 0.029) and HIITACT (p = 0.005). There were no significant differences in VO_2_ and HR between HIITPASS and HIITACT.

**Conclusions:**

The increase in HHb at VL and the lower mean power output during HIITACT could indicate that a higher level of deoxygenation contributes to decreased mechanical power in sedentary participants. The significant differences in HHb between sites indicates the specificity of oxygen utilisation.

## Introduction

Sedentary behaviour, defined as not meeting physical activity recommendations for the achievement of health benefits, is a risk factor for multiple chronic diseases [1, 2] and a global epidemic [1, 3–7]. Physical activity recommendations include accumulating 150–300 minutes of moderate intensity exercise each week [8]. The most frequently cited reason for non-compliance is a lack of time [9]. High intensity interval training (HIIT) of a low volume has been proposed as a time-efficient exercise format to improve exercise adherence, thereby reducing the chronic disease burden associated with sedentary behaviour [10]. The beneficial effect of HIIT interventions on markers of health risk has been well documented [11–13]. HIIT has been shown to be as effective or more effective than longer, moderate intensity exercise interventions at improving specific markers of risk, such as low cardiorespiratory fitness [11, 14, 15].

Benefits of regular HIIT exercise, such as increased cardiorespiratory fitness, have been linked to increases in mitochondrial content and function [16, 17]. Whilst the exact mechanisms underlying these increases are not completely understood, it is possible that the increase in oxygen utilisation at the local tissue level during an acute session of HIIT provides a stimulus for these improvements. The effects of a single HIIT intervention on systemic and locomotor muscle oxygenation have been evaluated previously [18, 19]. However, these investigations were conducted in active individuals, evaluated only one muscle site and used measures of oxygenation in a sports performance context [18, 20].

Site specific oxygen utilisation at the local tissue level can be measured using near infrared spectroscopy (NIRS). NIRS is a non-invasive method for the measurement of the change in concentration of oxyhaemoglobin (O_2_Hb) (oxygen availability) and deoxyhaemoglobin (HHb) (oxygen utilisation), as measures of tissue level oxygenation. Oxygen utilisation during exercise has been described in active individuals at a single muscle site [21] and in component muscles of the quadriceps [22, 23]. In active individuals, at a single muscle site, oxygen utilisation (as indicated by increased HHb) is increased during HIIT bouts when compared to pre-exercise values [18, 24].However, oxygen utilisation during HIIT in sedentary individuals at the local tissue level has not been determined. Furthermore it is unknown if oxygen utilisation differs between distinct locomotor muscles in a sedentary population during HIIT. Investigation of the oxygen utilisation responses in sedentary individuals will provide additional information on the extent to which oxygen utilisation increases during HIIT in distinct locomotor muscles, a potential stimulus for improved mitochondrial function.

Nine design components (series, inter-series, bout and recovery: number, duration and intensity as well as exercise mode) can be altered in HIIT [25]. The recovery periods of HIIT are an integral part of the exercise session, as these periods have an effect on the physiological responses that occur during the session [26].The two most frequently adopted HIIT recovery formats are passive and active recovery. The effect of recovery formats on local tissue oxygenation and markers of performance have yielded inconsistent findings to date, with active recovery leading to higher [18], lower [26] or an equivalent [24] degree of local muscle deoxygenation when compared with passive recovery. Similarly, inconsistent findings have been shown when mechanical power and heart rate were compared during HIIT that included either active or passive recovery [26–29]. Variations in the HIIT protocols used during these projects may have contributed to the conflicting results. The effect of active versus passive recovery periods on oxygen utilisation during HIIT bouts at specific locomotor muscle and brain tissue sites, in sedentary populations, is unknown.

The primary aim of this project was to compare the local (Δ[HHb]) and systemic (VO_2_) oxygen utilisation, mean power output and heart rate responses during HIIT conditions which included either passive or active recovery. A secondary aim was to compare the relative Δ[HHb] between local muscle and brain tissue sites during HIIT exercise.

It was hypothesised that in young sedentary individuals, during high intensity exercise bouts that are interspersed with active recovery periods, when compared to passive recovery periods, VO_2_, Δ[HHb],mean power output and heart rate would be higher and that the increase in Δ[HHb] during HIIT exercise would be higher at the local muscle tissue sites when compared to the brain site.

## Methods

### Ethics statement

This research project was approved by the human research ethics committee of the University of the Sunshine Coast (S/13/472). All participants received a research project information sheet before providing written informed consent.

### Experiment design

The project consisted of three testing sessions, one for each of the three conditions of the project. Exercise was performed using a cycling ergometer.

All testing sessions were separated by three to seven days to prevent a potential carry-over effect between conditions and to minimise the effect of any potential confounding variables between testing sessions. In this article, each 30 s period of high intensity exercise is referred to as a bout. Each complete protocol consisting of four x 30 s bouts of high intensity exercise, separated by 2 min recovery periods, is referred to as a condition.

**Fig 1:**
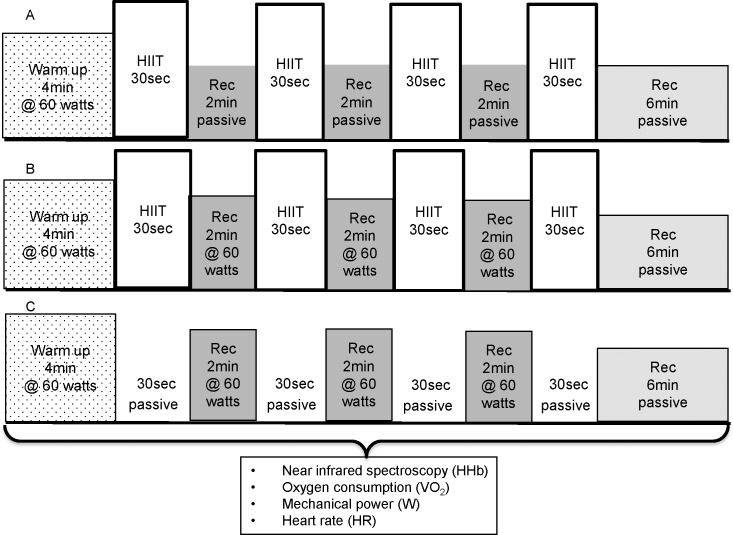
The structure and timing of measurements of the three conditions. (A) HIITPASS. (B) HIITACT. (C) REC.

The three conditions were: 1) a protocol of high intensity interval exercise with passive recovery periods between each bout of HIIT (HIITPASS) 2) a protocol of high intensity interval exercise with active recovery periods between each bout of HIIT (HIITACT) 3) a protocol in which only the active recovery periods were completed and the bouts of HIIT were replaced with passive periods (REC), in order to quantify the effect of active recovery. The conditions were randomized and followed a latin-squares cross-over design to control for a possible order effect. The conditions and the timing of measurements are illustrated in Fig 1.

### Participants

The participant group consisted of twelve males from the University community who met the inclusion criteria of being aged 18–30 yr.; currently completing less than 150 minutes of moderate intensity or 75 minutes of vigorous intensity activity per week; presenting with no cardiovascular and metabolic disease; taking no medications; having no known orthopaedic or other health related issues that would be made worse by participation in, or inhibit completion of the project. Descriptive physical characteristics of these participants are in Table 1.

**Table 1:** Participant characteristics.

Height (cm)	176.3 ± 8.3
Weight (kg)	78.19 ± 13.82
Age	23 ± 3
Vastus lateralis skinfold (mm)	12.75 ± 5.88
Gastrocnemius skinfold (mm)	11.00 ± 3.25
FVC (L)	5.40 ± 0.77
FVC % pred (%)	104.7 ± 10.2
FEV_1_ (L)	4.54 ± 0.72
FEV_1_% pred (%)	104 ± 10.9

Data are (mean ± SD) (n = 12)

### Procedures and equipment

#### Screening procedures

At the first testing session, participants completed risk screening and medical history questionnaires and a physical activity log. For the physical activity log participants reported the duration, intensity and type of activity which they had completed over the preceding seven days as well as the daily activity undertaken during an average week over the last three months. This physical activity log was used to ensure that participants’ recent activity levels were within the definition of sedentary for the purposes of this project (an individual not achieving the current minimal recommendations for exercise participation to gain health benefits) [30]. Participants were asked to refrain from performing any exercise in the 24 hours preceding each session and to not ingest any caffeine, alcohol or a large meal in the four hours preceding a session. Participants were asked to ensure that they were adequately fed and hydrated on the day of testing and this was confirmed at each testing session. To ensure normal resting pulmonary function, participants completed a pulmonary function test (Spirolab II, Medical International Research, Rome, Italy) following standard procedures [31] (Table 1). Participants were characterised by height, mass and adipose tissue thickness (ATT) (Table 1). ATT measurements, performed by the same researcher in each instance using skinfold callipers (Harpenden, British Indicators Ltd, Burgess Hill UK) and standard procedures, ensured that site-specific changes in oxygenation occurred within the muscle tissue rather than in the skin and adipose tissue.

#### Exercise conditions

Prior to the first exercise session, participants were familiarised with the Wingate testing protocol, the Velotron cycle ergometer (Racermate, Seattle WA, USA) and the process of holding a constant cadence. The cycle ergometer seat height and handlebar position were adjusted for each participant and replicated for subsequent exercise sessions.

The HIIT protocol utilised during two of the three conditions (HIITPASS and HIITACT) was adapted from protocols used in sporting, recreationally active and untrained populations. [20, 24, 32–36].

Each condition consisted of an initial baseline data collection period of 3 min when the participant remained stationary on the cycle ergometer. Exercise testing began with a 4 min warm up period. The warm up period consisted of each participant cycling against a fixed resistance of 60 Watts (W) at a cadence of 60 revolutions per minute (RPM). The warm up was followed by four 30 s bouts of high intensity exercise in the HIITPASS and HIITACT conditions, with 2 min recovery periods separating each of the high intensity bouts. During the REC condition, the four high intensity exercise bouts were replaced by periods of passive rest. Each participant was asked to increase cadence to a maximum during a five second period immediately preceding each bout of HIIT.

The resistance (0.075kg per kilogram body weight), automatically applied to the flywheel of the ergometer at the start of each bout of HIIT, was utilised during other HIIT Wingate protocols involving untrained adult populations [37]. Power output during the HIIT bouts was determined by participant effort. Participants were instructed to give a maximal effort from the beginning of each bout, using the prompt to ‘go as hard as you can’. Participants were then verbally encouraged using standardised phrases during all bouts of exercise in an attempt to ensure a maximal effort. During the passive recovery periods of the HIITPASS condition, participants were instructed to sit as still as possible with the bicycle cranks in a relaxed horizontal position. During the active recovery periods of the HIITACT and REC conditions, participants were instructed to pedal at a cadence of 60 RPM against a resistance of 60 W (approximately 30–40% VO_2max_) [38], an intensity of active recovery shown to promote optimal clearance of metabolites [39, 40]). Upon completion of the fourth and final bout, there was a 6 min passive recovery period.

Participants were instructed to remain seated throughout each condition in an attempt to reduce movement artefact in the NIRS data and to allow for consistency in muscular recruitment patterns and hence power data.

#### Tissue oxygenation

Changes in local tissue oxygenation were measured continuously during rest, exercise and recovery. The terms oxygenated haemoglobin (O_2_Hb), and deoxygenated haemoglobin (HHb) each include the combined signal of Hb and myoglobin (Mb). The changes in the relative concentration of O_2_Hb (Δ[O_2_Hb]) and HHb (Δ[HHb]) as a function of time were measured using a Near Infrared Spectroscopy (NIRS) system (2 x PortaMon and 2 x Portalite devices, Artinis Medical Systems BV, Zetten, Netherlands). This system allows for non-invasive and simultaneous measurement of these variables at multiple sites. The NIRS system uses a modified form of the Beer-Lambert law to calculate changes in O_2_Hb and HHb using two continuous wavelengths of near infrared light (763 and 855nm). A fixed differential pathlength factor (DPF) of 4 was used for muscle tissue and an age dependant DPF was used for cerebral tissue based on manufacturer recommendations.

The NIRS devices (weighing 84 grams with dimensions of 83 x 52 x 20 mm) were placed on the shaved skin overlying the muscle belly of two locomotor muscles of the left leg, the vastus lateralis (VL) and the gastrocnemius (GN), a muscle involved in respiration: the 7th external intercostal muscle (IC) and the area of the forehead overlying the pre-frontal cerebral cortex (FH) approximately 3 cm left from the forehead midline and immediately above the supra-orbital ridge (between Fp1 and F3, according to the modified international EEG 10–20 system). To ensure measurement consistency, the placement of the NIRS devices was referenced to accepted anatomical landmarks as detailed in previous experiments [18, 24, 41–43]. The location of a devices was marked with a felt tip pen at the first testing session and participants were instructed to maintain these marks between sessions. Each device was secured using standardised procedures to shield against ambient light contamination and to prevent motion artefact due to device slippage. For all testing the same device was used at the same measurement site for each participant. The NIRS system was connected via Bluetooth to a computer for data acquisition and subsequent data analysis.

**Fig 2:**
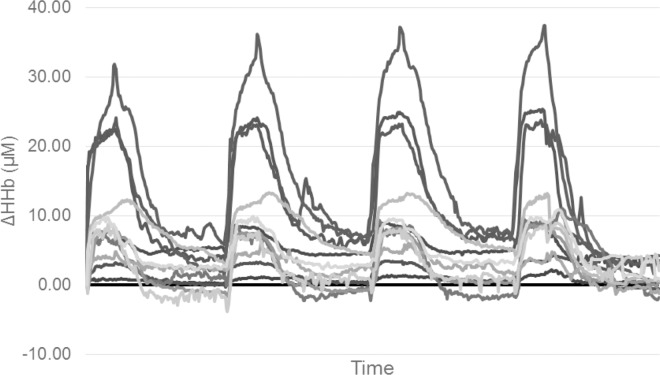
Individual relative change from baseline of deoxygenated haemoglobin (HHb) during the HIITACT condition. (n = 12).

For this project, both Δ[O_2_Hb] and Δ[HHb] were measured, however only Δ[HHb] values are presented. The Δ[HHb] data are potentially unaffected by changes in perfusion, blood volume or arterial haemoglobin concentration [44–46]. The Δ[O_2_Hb] data are affected by muscular compression and changes in blood flow and volume [47], especially during the rapid and substantial changes in these variables that accompany HIIT bouts [18]. Using Δ[HHb] is consistent with other research utilising NIRS measurements to investigate HIIT and exercise in general [18, 24] thereby allowing for comparisons to be made between this project and previous research. NIRS data collected at the IC site included gross movement artefact throughout testing, obscuring the NIRS signal. Gross movement artefact was also present in NIRS data collected at the VL and GN sites during the passive recovery periods of the HIITPASS condition. Therefore data collected at the IC site and recovery period data from other sites were not included in further analysis. The inter-individual variability noted in the Δ[HHb] data (range 0.73–32.56 μM) is presented for the VL in HIITACT (Fig 2).

#### Systemic oxygen consumption (VO_2_)

In order to quantify the systemic oxygen utilisation in response to the three conditions, systemic oxygen consumption data was collected continuously during the rest, exercise and recovery periods using a respiratory gas analysis open circuit spirometer system (Parvo Medics, Sandy UT, USA) and a standard gas collection mouthpiece (Hans Rudolph, Kansas, United States of America). Standardised calibration and methods were used [48].

#### Mechanical power

Mechanical power was measured during the cycling portions of each condition using a SRM ‘Science’ power meter (SRM, Julich, Germany). Prior to each testing session the SRM unit was calibrated according to the manufacturer’s specifications.

#### Heart rate (HR)

To quantify exercise intensity during all exercise conditions, a heart rate monitor (RS400, Polar Electro, Kempele, Finland) was used to measure heart rate data during rest, exercise and recovery periods.

### Data calculation and statistical analysis

All NIRS data were collected at a frequency of 10 Hz and smoothed using a 10 point moving average before being averaged to 1 s periods. Due to the HHb data being a measure of change from an arbitrarily assigned baseline zero value, the NIRS data are expressed as units of change (μMol) from the mean value of the 30 s of baseline data preceding the start of exercise (Δ[HHb]). To determine the between test reliability of the HHb data from the NIRS system, absolute reliability (Typical Error: VL = 0.4, GN = 0.8, FH = 0.6) of the baseline data for each site was used. Furthermore, previous research has shown that the NIRS method provides acceptable reliability [49, 50]. VO_2_ data were averaged over 5 s periods in preparation for further analysis whilst mechanical power and HR data were averaged at 1 s intervals. The NIRS, HR, VO_2_ and power data were then time aligned and the time periods of data corresponding to the four 30 s bouts of HIIT identified. Mean 30 s values were then calculated for all dependant variables for each bout of HIIT, providing a single value per bout for statistical analysis.

#### Statistics

Statistical tests were performed using IBM SPSS Statistics (version 22, IBM Corporation, Armonk NY, USA). Data was initially screened for normality of distribution using a Shapiro-Wilk test. A two factor, repeated-measures analysis of variance (ANOVA) was used to analyse the effect of condition and bout on the dependant variables of VO_2_, HR and mechanical power. A three factor, repeated-measures ANOVA was used to analyse the effect of condition, bout and site on the dependant variable Δ[HHb]. Mauchly’s W test was used to evaluate sphericity for each dependant variable. For those variables that violated the assumption of sphericity, the degrees of freedom were adjusted using the Greenhouse-Geisser correction if the estimate of sphericity (є) < 0.75. If є >0.75 the Huynh-Feldt adjustment was used and significance re-evaluated. If a significant main effect was identified, a Bonferroni’s post hoc test was used to make pair wise comparisons. All variables are presented as mean ± standard deviation (SD). For all statistical analyses, a P value of < 0.05 was accepted as the level of significance.

## Results

### Tissue oxygenation

For the mean Δ[HHb] from all conditions, sites and bouts combined, there was a main effect for site (p = 0.003, F = 7.493), with significant differences found between FH and VL sites (p = 0.025) and the difference between GN and VL sites approaching significance (p = 0.056). There was also a main effect for condition (p < 0.001, F = 20.899), however no significant differences were found between the two HIIT conditions. There were condition x site (p = 0.016) and site x bout (p = 0.002) interactions.

For the mean Δ[HHb] for each condition, there was a main effect for site for HIITPASS [(p = 0.029, F = 4.346) FH = 1.97 ± 2.69 μM, GN = 4 ± 4.46 μM, VL = 6.97 ± 5.42 μM] and HIITACT [(p = 0.005, F = 10.014) FH = 1.81 ± 2.65 μM, GN = 4.44 ± 3.75 μM, VL = 10.722 ± 8.48 μM] with significant differences found between FH and VL (p = 0.018) and GN and VL (p = 0.035) for HIITACT. There were site x bout interactions for HIITPASS (p = 0.001) and HIITACT (p = 0.042). No significant differences were found for REC [FH = -0.28 ± 1.16 μM, GN = -0.64 ± 2.39 μM, VL = 0.22 ± 3.38 μM].

#### Forehead (FH)

For the FH there was a main effect in the mean Δ[HHb] for condition, however no significant differences were found between the two HIIT conditions: [(p = 0.002, F = 8.513) HIITPASS 1.97 ± 2.69 μM; HIITACT 1.8 ± 2.65 μM; REC -0.27 ± 1.16 μM]. There was a condition x bout interaction (p = 0.003).

**Fig 3:**
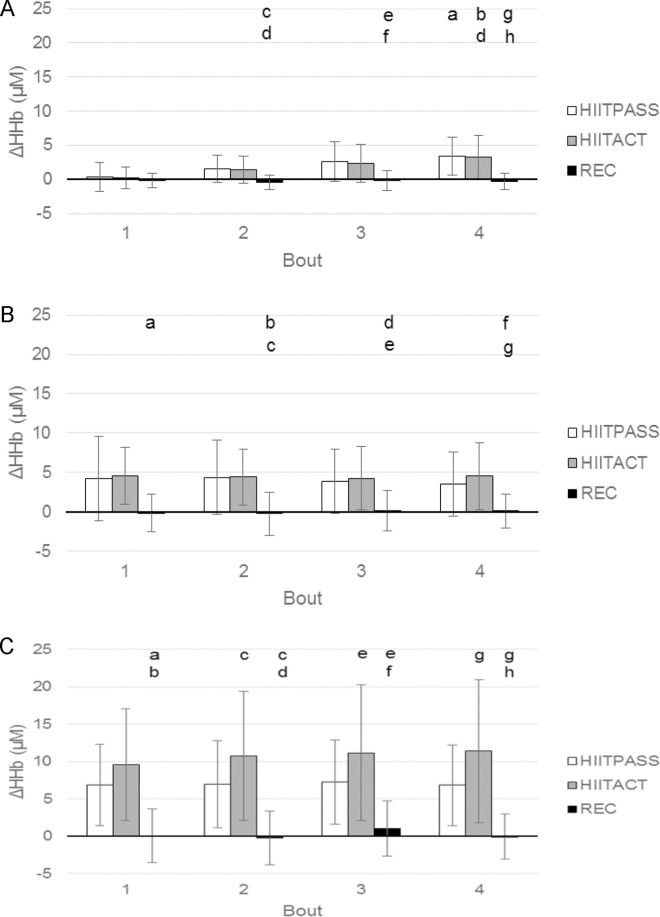
Relative change from baseline of deoxygenated haemoglobin (HHb) concentration during the four bouts of the three conditions. (A) FH. a = significantly different to HIITPASS, bout 1; b = significantly different to HIITACT, bout 1; c = significantly different to HIITPASS, bout 2; d = significantly different to HIITACT, bout 2; e = significantly different to HIITPASS, bout 3; f = significantly different to HIITACT, bout 3; g = significantly different to HIITPASS, bout 4; h = significantly different to HIITACT, bout 4. (B) GN. a = significantly different to HIITACT bout 1; b = significantly different to HIITPASS, bout 2; c = significantly different to HIITACT bout 2; d = significantly different to HIITPASS, bout 3; e = significantly different to HIITACT bout 3; f = significantly different to HIITPASS, bout 4; g = significantly different to HIITACT, bout 4. (C) VL. a = significantly different to HIITPASS, bout 1; b = significantly different to HIITACT, bout 1; c = significantly different to HIITPASS, bout 2; d = significantly different to HIITACT, bout 2; e = significantly different to HIITPASS, bout 3; f = significantly different to HIITACT, bout 3; g = significantly different to HIITPASS, bout 4; h = significantly different to HIITACT, bout 4. Data are mean ± SD. (p >0.05).

For the mean Δ[HHb] for each bout (Fig 3, panel A), differences were found between conditions for Bout 2 (p = 0.006, F = 6.504), Bout 3 (p = 0.003, F = 7.845) and Bout 4 (p < 0.001, F = 12.758), however no significant differences were found between the two HIIT conditions. For the mean Δ[HHb] within conditions, there were significant increases across bouts, with values increasing over time in the HIITPASS (p = 0.003, F = 9.733) and HIITACT (p = 0.007, F = 8.511) conditions.

#### Gastrocnemius (GN)

For the GN there was a main effect in the mean Δ[HHb] for condition, however no significant differences were found between the two HIIT conditions: [(p < 0.001, F = 11.911) HIITPASS 4.00 ± 4.46 μM; HIITACT 4.4 ± 3.75 μM; REC -0.06 ± 2.39 μM]. There was no significant condition x bout interaction.

For the mean Δ[HHb] for each bout (Fig 3, panel B), differences were found between conditions for Bout 1 (p = 0.006, F = 9.270), Bout 2 (p < 0.001, F = 11.339), Bout 3 (p = 0.001, F = 9.475) and Bout 4 (p < 0.001, F = 12.765), however no significant differences were found between the two HIIT conditions. For the mean Δ[HHb] within conditions, no significant differences were found across bouts.

#### Left vastus lateralis (VL)

For the VL, there was a main effect in the mean Δ[HHb] for condition: [(p = 0.003, F = 13.060) HIITPASS 6.97 ± 5.42 μM; HIITACT 10.72 ± 8.48 μM; REC 0.22 ± 3.38 μM]. There was no significant condition x bout interaction.

For the mean Δ[HHb] for each bout (Fig 3, panel C), differences were found between conditions for Bout 1 (p = 0.002, F = 14.835), Bout 2 (p = 0.003, F = 12.968), Bout 3 (p = 0.006, F = 10.587) and Bout 4 (p = 0.004, F = 12.575) with significant differences found between the two HIIT conditions. For the mean Δ[HHb] within conditions, there were no significant differences found across bouts.

### Systemic oxygen consumption

For the mean VO_2_, there was a main effect for condition [(p < 0.001, F = 161.601), however no significant differences were found between the two HIIT conditions [HIITPASS 29.6 ± 3.26 ml.kg.min^-1^; HIITACT 31.4 ± 4.53 ml.kg.min^-1^; REC 12.3 ± 1.61 ml.kg.min^-1^].There was no significant condition x bout interaction.

**Fig 4:**
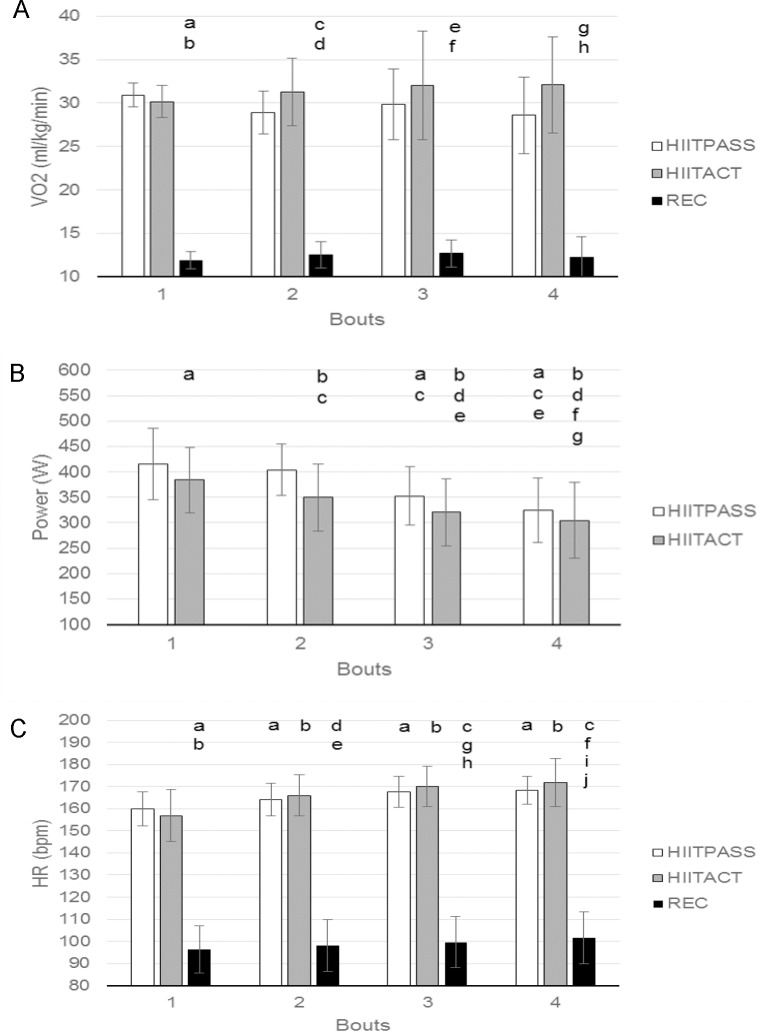
Oxygen consumption, mechanical power and heart rate during the four bouts of the three conditions. (A) VO_2_. a = significantly different to HIITPASS, bout 1; b = significantly different to HIITACT, bout 1; c = significantly different to HIITPASS, bout 2; d = significantly different to HIITACT, bout 2; e = significantly different to HIITPASS, bout 3; f = significantly different to HIITACT, bout 3; g = significantly different to HIITPASS, bout 4; h = significantly different to HIITACT, bout 4. (B) Mechanical power. a = significantly different to HIITPASS, bout 1; b = significantly different to HIITACT, bout 1; c = significantly different to HIITPASS, bout 2; d = significantly different to HIITACT, bout 2; e = significantly different to HIITPASS, bout 3; f = significantly different to HIITACT, bout 3; g = significantly different to HIITPASS, bout 4; (C) HR. a = significantly different to HIITPASS, bout 1; b = significantly different to HIITACT bout 1; c = significantly different to REC, bout 1; d = significantly different to HIITPASS bout 2; e = significantly different to HIITACT, bout 2; f = significantly different to REC, bout 2; g = significantly different to HIITPASS, bout 3; h = significantly different to HIITACT, bout 3; i = significantly different to HIITPASS, bout 4; j = significantly different to HIITACT, bout 4. Data are mean ± SD. (p >0.05).

For the mean VO_2_ for each bout (Fig 4, panel A), differences were found between conditions for Bout 1 (p < 0.001, F = 491.444), Bout 2 (p < 0.001, F = 161.961), Bout 3 (p < 0.001, F = 77.016) and Bout 4 (p < 0.001, F = 72.269), however no significant differences were found between the two HIIT conditions. For the mean VO_2_ within conditions, no significant differences were found across bouts, with values remaining relatively similar over time.

Mean VO_2peak_ within conditions and across bouts had the same statistical differences as mean VO_2_.The differences in VO_2_ when comparing the REC condition to the HIITPASS and HIITACT conditions were as expected (Fig 4 panel A).

When total oxygen consumption was compared across the three conditions, differences were found [(p < 0.001, F = 78.562) HITTPASS 12.7 ± 2.06 litres; HIITACT 16.0 ± 3.02 litres; REC 8.4 ± 0.79 litres] with significant differences between all conditions.

### Mechanical power

For the mean power output, there was a main effect for condition [(p < 0.001, F = 57.636). HIITPASS 374.3 ± 70 W; HIITACT 339.9 ± 72.7 W]. There was no significant condition x bout interaction.

For the mean power output for each bout (Fig 4, panel B), differences were found between conditions for Bout 1 (p = 0.026, F = 6.612), Bout 2 (p < 0.001, F = 87.513), Bout 3 (p < 0.001, F = 24.144) and Bout 4 (p = 0.045, F = 5.118). For the mean power output within conditions, decreases were found across bouts over time (HIITPASS p < 0.001, F = 25.281; HIITACT p < 0.001, F = 22.923).

### Heart rate

For the mean HR there was a main effect for condition [(p < 0.001, F = 395.034), however no significant differences were found between the two HIIT conditions: HIITPASS 165 ± 7.6 bpm. HIITACT 166 ± 11.5 bpm. REC 99 ± 11.3 bpm]. There was a condition x bout interaction (p = 0.022)

For the mean HR for each bout (Fig 4, panel C), differences were found between conditions for Bout 1 (p < 0.001, F = 224.796), Bout 2 (p < 0.001, F = 375.081), Bout 3 (p < 0.001 F = 417.149) and Bout 4 (p < 0.001, F = 289.073) however no significant differences were found between the two HIIT conditions. For the mean HR within conditions, increases were found across bouts over time. HIITPASS (p < 0.001, F = 20.234); HIITACT (p < 0.001, F = 16.574); REC (p < 0.001, F = 14.227).

Average HR_peak_ within conditions and across bouts provided the same statistical differences as mean HR. The differences in HR when comparing the REC condition to the HIITPASS and HIITACT conditions were as expected (Fig 4, panel C).

## Discussion

The primary aim of this project was to compare the local (Δ[HHb]) and systemic (VO_2_) oxygen utilisation, mean power output and heart rate responses during HIIT conditions which included either passive or active recovery. A secondary aim was to compare the relative Δ[HHb] between local muscle and brain tissue sites during HIIT exercise. In support of our hypotheses, there was a significant increase in HHb at the VL site during the 2^nd^, 3^rd^ and 4^th^ high intensity exercise bouts interspersed with active recovery periods (HIITACT) when compared to bouts interspersed with passive recovery periods (HIITPASS). Also supporting our hypotheses, when including the exercise performed during the active recovery periods, the total VO_2_ was significantly higher during the HIITACT condition when compared to both the HIITPASS and REC conditions. In opposition to our hypotheses, mean power output during the exercise bouts was significantly higher in the HIITPASS condition when compared to the HIITACT condition. No significant differences were found in Δ[HHb] at the (FH) and (GN) sites, in mean VO_2_ and in mean HR during the high intensity exercise bouts when comparing HIITPASS and HIITACT. There were significant differences for all dependant variables when comparing the bouts in REC to HIITPASS and HIITACT. These results were expected and validate the use of the REC protocol as a control condition.

### Tissue oxygenation

The significantly higher Δ[HHb], indicating increased oxygen utilisation, at the VL site during the second, third and fourth bouts of the HIITACT condition, when compared to the HIITPASS condition could indicate a response to the reduced reoxygenation of the muscle during the active recovery portions, leading to a higher deoxygenation of the muscle tissue during subsequent bouts. This would explain the significantly decreased mean power outputs that occur during the HIITACT bouts, as increased deoxygenation would impair subsequent performance in this large locomotor muscle, potentially due to impaired phosphocreatine (PCr) resynthesis linked to competition for limited oxygen resources [38] or via centralised neuromuscular downregulation in response to the increased rate of biochemical changes [51]. In intermittent sprints [18, 24] the same pattern of reduced performance measures and higher levels of deoxygenation during active recovery conditions has occurred, if insufficient time is provided for a complete physiological recovery [26, 29]. To the contrary, Calbet et al [52] suggest that there may be a functional reserve in oxygen diffusing capacity during exercise, in which situation the higher level of deoxygenation observed during the HIITACT condition would not be considered limiting. Due to differences in the design of the experiments it is difficult to compare the findings of these projects, however it is possible that the oxygen utilisation at the VL site, which is the primary locomotor muscle for cycling [53], is likely to be underestimated by the assessment of whole leg oxygen utilisation via femoral blood samples by Calbet et al.

Additionally, the significantly higher Δ[HHb] in the VL during the bouts of the HIITACT condition cannot be accounted for by the effect of active recovery as a simple additive process (i.e. HIITPASS + REC = HIITACT), as the differences in the Δ[HHb] between the bouts of the two HIIT conditions are higher than the Δ[HHb] during the bouts of the REC condition (Fig 3C).

In the smaller GN muscle of the same leg, which has a lesser role in power production during cycling [53] and a greater percentage of oxidative muscle fibres [54, 55], there were no significant differences in Δ[HHb] between conditions. During exercise, the muscle with a greater percentage of oxidative fibres (GN) would be able to meet the increasing energy requirements, creating little change in HHb values, compared to pre-exercise values. The reasons provided above could also explain why the magnitude of mean Δ[HHb] in the VL muscle was greater than that in the GN muscle in both the HIITPASS and HIITACT conditions.

When comparing Δ[HHB] within conditions, there was no progressive muscle deoxygenation observed in VL and GN from bouts one to four irrespective of recovery type. If exercise effort was maximal, in line with participant instructions to give a maximal effort during each bout, an upper oxygen utilisation limit was reached during each effort at both muscle sites. This has occurred in previous research involving repeat Wingate testing [24]. When the significant reductions in mechanical power are taken into consideration, this indicates that declining muscle performance is associated with repeated maximal levels of oxygen utilisation, providing evidence that maximal oxygen utilisation may not be a limiting factor during HIIT, in sedentary participants. Assessment of the rate of muscle deoxygenation and reoxygenation could yield important information to further delineate differences between HIIT conditions and provide additional information into potential mechanisms involved in site specific oxygen utilisation. Whilst an important future research direction, this assessment was not possible during the current project due to artefact present in the NIRS data during recovery portions of the PASSHIIT condition and the impracticality of cuff occlusion (a common practice when examining deoxygenation and reoxygenation rates [56]) during supramaximal intermittent exercise whilst measuring HHb in multiple limb segments.

The inter individual variability in the VL Δ[HHb] response during the HIITACT condition (Fig 2) was unrelated to the participants power output (i.e. the participants with the highest power outputs did not show the greatest increases in HHb). Additionally, mean Δ[HHb] remained unchanged over time whilst mean power decreases significantly, hence the variability cannot be explained by a simple demand-driven system. A similar degree of variability in the Δ[HHb] response can be seen in the one project to publish individual results [57] or by noting the standard deviation of the Δ[HHb] signal if the method of data calculation and analysis is similar [58]. Currently there is no standard for NIRS instruments or for the method of calculating, analysing and presenting NIRS data [59]. This makes comparison of Δ[HHb] data between projects difficult, even when projects have been performed in similar populations, performing similar HIIT interventions. This in turn limits the ability of researchers to gain a comprehensive understanding of what constitutes a normal NIRS response (or range) during exercise in sedentary populations.

When comparing the FH Δ[HHb] from bout one to bout four, HHb concentrations did not rise significantly until the last bout of both HIIT conditions. This response could be due to the brain being protected from homeostatic disturbances and therefore adequately perfused during the majority of exercise bouts [51]. Late in maximal exercise cerebral vasoconstriction, diminished cerebral blood flow and an increase in cerebral oxygen uptake occur [60, 61]. These factors would explain the late rise in HHb. A similar response is noted in previous intermittent sprint research [42, 62].

Interpretation of differences in the Δ[HHb] data between sites should be made with caution. A significant main effect for site was found, however the NIRS devices measure relative Δ[HHb] from an arbitrary baseline. Parameters that potentially affect NIRS measures, such as blood flow and muscle tension, are not routinely measured in conjunction with HHb. Therefore a lack of significant differences in the Δ[HHb] between the FH and GN sites does not necessarily indicate that the oxygen utilisation responses to the exercise stimulus are the same at these two sites.

### Systemic oxygen consumption

Previous research [63] has indicated that active recovery is associated with increased oxygen consumption during exercise bouts when compared to passive recovery. However, no significant differences were found for the HIITPASS and HIITACT conditions when comparing mean VO_2_ between conditions and across bouts. Our findings are in agreement with other research [29], although that study incorporated open ended intermittent exercise. Potential mechanisms for no difference between conditions could include compensatory decreased oxygen utilisation at non-exercise associated tissue.

The lack of differences across bouts was unexpected, since the response in each subsequent exercise bout is not an isolated exercise period, but would be expected to be, in part, a function of the previous bouts. Therefore an increase in VO_2_ and HR as a function of time was predicted due to incomplete physiological recovery in the 2 min available to the sedentary individuals between supramaximal exercise bouts. A potential explanation for no difference across bouts could be, if oxygen utilisation is maximised at the muscle (as indicated by the relatively stable Δ[HHb] over time in both VL and GN) a similar pattern would be expected in systemic oxygen utilisation measured at the mouth, and any differences in work across bouts would be met anaerobically. Other potential contributing factors to the results obtained include the significant decrease in mechanical power output across bouts, effectively requiring less aerobic contribution over time, in effect counteracting the increased VO_2_ due to the cumulative load of the protocol. Furthermore, whilst we did not measure maximal VO_2_ in this group of participants, it is conceivable that VO_2_ values in excess of 30 ml.kg.min^-1^ were maximal in these sedentary individuals, effectively creating a ceiling effect in each and every bout.

### Mechanical power

The mean mechanical power achieved during the exercise bouts was lower than that achieved by active individuals performing repeat Wingate tests [64]. Mean mechanical power declined significantly from the first to the fourth bout in both the HIITPASS and HIITACT conditions. This was expected, due to the cumulative fatigue and incomplete ATP repletion and PCr resynthesis that occur during repeat Wingate exercise that include recovery periods that do not allow for complete recovery [24, 28, 65].

The HIITACT bouts were all performed at a lower mean power output when compared to the HIITPASS bouts. To the best of our knowledge no similar HIIT research has been done in sedentary individuals. However, in active populations when comparing the effect of active and passive recovery on mean power output during intermittent sprints, contradictory results have been published. Some researchers found no significant differences [65–67], whilst others have found that passive recovery protocols yielded greater mean power values [24]. Lopez et al [28] found that an active recovery condition resulted in a significantly greater mean power output in later (5^th^ and 6^th^) Wingate bouts. This finding could be explained by the fact that the longer the exercise session, the greater the contribution of active recovery to metabolite clearance, the correction of a cellular acidosis and resynthesis of PCr, which enabled a lower magnitude decline in mean power production in the later Wingate bouts.

The contradictory findings above could also be explained by differences in the intensity of active recovery utilised in different projects, highlighting the difficulty in comparing HIIT research due to the variety available when designing exercise protocols.

Current evidence suggests that passive recovery can improve subsequent performance when recovery duration is short (15–120 seconds) and / or exercise intensity is high [26]. Our findings in sedentary individuals support this. Choosing an appropriate recovery format (and duration) is an important consideration when considering prescription of HIIT to time poor sedentary individuals, as recovery periods would be of relatively short duration to ensure that the entire HIIT session is in fact shorter than the current moderate intensity exercise recommendations.

The lower mean power output in the HIITACT condition occurred from the first bout. In a sedentary population with no previous experience of repeat Wingate testing, participants potentially adopted a subconscious feedforward pacing strategy to minimise the ‘additional’ discomfort associated with a condition including an active recovery.

In each subsequent bout, mean power output was significantly lower in the HIITACT condition compared to the HIITPASS condition. The local muscle recovery includes the resynthesis of PCr, which relies on oxygen dependant pathways and is strongly correlated with repeat sprint ability [68]. Competition for available oxygen supplies may occur between the processes of PCr resynthesis, lactate oxidation and the oxygen cost of continued exercise itself during active recovery [26, 28, 38, 69, 70]; causing a decrease in performance and power generation when active recovery protocols are adopted. Additionally, in sedentary populations the improved active recovery clearance of metabolites may not occur in an acute session of HIIT due to the fact that the improved clearance of metabolites is an adaptation that occurs with routine exercise training at higher intensities [71]. Due to the untrained state of participants, passive rest periods may allow participants to recover to a greater extent, enabling a lesser reduction in power output over the entirety of the HIIT session.

### Heart rate

In both HIIT conditions mean HR rose significantly when comparing bout one to later bouts, in line with findings from previous research [28].

In opposition to previous research [28, 65, 67] showing an active recovery condition is associated with a greater HR response when compared to a passive recovery condition, no significant differences were found between the HIITPASS and HIITACT conditions for mean HR overall and during each bout. This response is however in agreement with other research [29]. The lack of difference found in our project could be explained by the significantly lower power output generated by the sedentary participants during the HIITACT condition. The lower power output could be responsible for a lesser HR response during the HIITACT bouts even though the active recovery portions of the condition kept HR higher during active recovery. During the HIITPASS condition, a higher power output would cause a greater rise in HR, but from a lower starting point due to a greater HR recovery during the passive recovery portions, in effect providing no difference in mean HR during each bout when comparing across conditions.

## Limitations

The participants within this group, whilst relatively homogenous in their sedentary behaviour during the project, were heterogeneous in their past exercise behaviours. This variability could potentially have confounded results. Participants needed to maintain a high level of motivation during the repeated maximal HIIT bouts. Individuals with extensive past exercise experience, even if currently sedentary, could rely on past exercise experience to better cope with the inherent discomfort of HIIT. Those with little or no previous exercise experience would potentially adopt either conscious or subconscious pacing strategies in an attempt to reduce the discomfort associated with repeated all-out efforts, despite instructions to perform maximally.

A number of participants reported feelings of dizziness and nausea during the recovery period of the HIITPASS condition. Although these symptoms were sub-clinical in severity, the negative sensations could have impacted on the participant’s exercise behaviours within the study.

Cycling was chosen for the current project due to practical and safety considerations. However, choosing a mode of exercise with a high degree of specificity may have limited the performance of sedentary individuals who would be more accustomed to weight-bearing forms of ambulation. The effect of mode on performance during HIIT in sedentary individuals requires further investigation.

## Conclusions

During the HIITACT condition a higher level of deoxygenation in the VL muscle and a lower mean power output occurred, when compared to the HIITPASS condition. This suggests that the higher level of deoxygenation could have contributed to an impaired performance during the HIITACT condition.

However, when compared across bouts within each condition, the level of deoxygenation did not change significantly at either the VL and GN sites from bout one to bout four, when participants were instructed to give a maximal effort. This indicates that oxygen utilisation reached maximal (but different) values at the two sites in each bout. Mean power output decreased over the course of both conditions. It can therefore be concluded that maximal oxygen utilisation is not a limiting factor in sedentary individuals over the course of a HIIT condition, irrespective of the inclusion of active or passive recovery periods.

The level of deoxygenation at the FH site did not increase significantly from pre-exercise values until the fourth bout in both HIIT conditions. This suggests that cerebral oxygenation was adequate until late in supramaximal exercise, possibly due to the importance of maintaining adequate cerebral perfusion.

To our knowledge this is the first research to show that in sedentary participants, the Δ[HHb] levels attained during HIIT exercise varies in different locomotor muscles of the same leg, indicating the specificity of individual muscle oxygen utilisation.
